# The relationship between climate classes and particulate matters over Europe

**DOI:** 10.1038/s41598-024-80365-7

**Published:** 2024-11-21

**Authors:** Jure Pražnikar

**Affiliations:** Faculty of Mathematics, Natural Sciences and Information Technologies, University of Primorska, Glagoljaška 8, Koper, SI-6000 Slovenia

**Keywords:** Climate-change impacts, Projection and prediction, Environmental impact

## Abstract

Present and future Köppen-Geiger climate maps and time series of particulate matter (PM10) at continental scale were used to investigate the relationship between spatial patterns of PM10 and climate zones. Five main patterns (spatial clusters) of PM10 were found over the European continent, which show a good spatial overlap with the main Köppen-Geiger climate zones. The map of future climate, which shows a poleward movement of the warmer climate zones, an expansion of the (semi-)arid zones and a shrinking of the polar and tundra zones, indicates a higher PM10 concentration, especially in the northern part of Europe. The results also show that there is a temporal shift (later/earlier) of PM10 extremes in the period 2013–2022. This insight into the relationship between climate zones and clustered PM10 time series and the use of high-quality future climate maps based on different scenarios can be used to estimate possible changes, such as annual averages or seasonal cycles, in PM10 concentrations.

**Supplementary Information:**

The online version contains supplementary material available at 10.1038/s41598-024-80365-7.

## Introduction

The Köppen-Geiger classification is one of the most frequently used methods for classifying the Earth’s climate zones^1^. This classification is based on monthly precipitation and temperature data, which are used to determine climate classes that share common vegetation characteristics. The distribution of climate classes on Earth shows some clear-cut boundaries between them, which is directly related to the sensitivity of plants to temperature and precipitation. In addition, climate maps^2–7^ can also be used to identify biomes and soil types^8^. The Köppen-Geiger classification is not only useful for studying the past^9^ and the present, but also allows us to examine potential impacts on vegetation, health^10,11^ and pollution in the future.

It has been shown that global climate change could have a major impact on the world’s population^12^. Due to rising temperatures, projections show that temperate climates are likely to be replaced by tropical and arid climates, and drier climate subtypes could spread to higher altitudes. All these shifts in climate zones are likely to affect up to 21% of the world’s population, or more than 1.5 billion people. It should be emphasized that the effects of changing climate zones on people, the economy, health and vegetation can be both positive and negative. In a subarctic climate, for example, milder winters and longer summers can have a positive impact on agriculture. On the other hand, the expansion of the arid climate, which is reflected in longer periods of drought or more extreme weather situations, can significantly worsen living conditions for most living organisms and endanger entire ecosystems.

Several studies have shown that climate change can also increase the concentration of air pollutants^13–16^. Longer dry periods and weaker wet deposition of particulate matter can potentially lead to higher concentrations of particulate matter, black carbon and carbon monoxide^17^. Although studies show a global increase in particulate matter pollution due to climate change, the predicted concentrations could also decrease locally^18^. Due to climate change, which relates to the amount, distribution and frequency of precipitation, the rise in temperature and the shift in weather patterns, it is important to increase knowledge about the relationship between particulate matter concentrations and climate zones.

Previous studies already suggest that particulate matter time series can be divided into groups – spatial patterns - that have also been associated with climate zones^19,20^, but these studies were conducted with data from land-based monitoring stations and over a relatively short period of time (3 years). Furthermore, the distribution of land-based stations is not homogeneous and only a very limited number of stations are continuously in operation over a period of 10 years. As a result, there is a lack of data, of in-situ measurements, particularly for the Balkan region, south-eastern Europe and eastern Europe. The lack of data is not least due to whether the country is a member of the European Union. In addition, station density is highest where the population is densest, as the data is needed to study the relationship between air pollution, mortality and disease, and there are only a limited number of land-based stations to measure air pollution in unpopulated regions, especially at high altitudes. To fill the gap of unevenly distributed land-based stations and to analyze data over a longer period of time, advanced forecasting models can be used instead.

The aim of this study is to use 10-year data from the Copernicus Atmosphere Monitoring Service (CAMS) Europe Air Quality Reanalyses, which provide good spatial and temporal coverage, to analyze the relationship between particulate matter time series and climate zones. Thus, the Köppen-Geiger climate zones map was used to investigate the spatial overlap between the present climate zones and the particulate matter time series and to investigate the seasonal trends, while the future climate zones map was used to qualitatively estimate the shift (decrease/increase) in particulate matter concentrations. This study provides additional insights into the relationship between Köppen-Geiger climate zones and particulate matter. It also expands the knowledge about possible changes in particulate matter concentrations on a continental scale due to climate change.

## Methods

### Data

Particulate matter (PM10) data from the CAMS Europe Air Quality Reanalyses were analyzed. The data are available at: https://ads.atmosphere.copernicus.eu. The data were available in a 0.1° x 0.1° longitude and latitude grid on the regional out-grid domain (-25 W/45E/30 N/70 N). The ensemble median based on the median method of predictions from seven atmospheric chemistry models^21^ was used. Daily mean PM10 concentrations were calculated using hourly data. For this study, the daily mean surface concentration from January 1, 2013 to December 31, 2022 was used, for which validated reanalyses from 2013 to 2020 and interim reanalyses 2021–2022 were available.

The Köppen-Geiger classification data were taken from the published paper Present and future Köppen-Geiger climate classification maps at 1-km resolution^7^. available at: https://www.gloh2o.org/koppen/. The present (1980–2016) map was derived from an ensemble of four high-resolution, topographically corrected climate maps, while the future (2071–2100) map was derived from an ensemble of 32 climate model projections - scenario RCP8.5^22^. The future map (2050) - scenario RCP4.5 was taken from Cui et al., 2021^2^, available at http://glass.umd.edu/KGClim/.

### Preprocessing of data

The data was preprocessed before an unsupervised machine learning technique (clustering) was performed. To enable a comparison between the Köppen-Geiger climate zones and the PM10 data, only land grids from the regional out-grid domain were selected. The Global Self-consistent, Hierarchical, High-resolution Geography Database, available at https://www.soest.hawaii.edu/pwessel/gshhg/, was used to define the coastline. To reduce RAM memory requirements, the original data (0.1° x 0.1°) of PM10 was remapped to a horizontal resolution of 0.2° x 0.2°. The Köppen-Geiger future and present data (0.083° x 0.083°) were also remapped to the same horizontal resolution (0.2° x 0.2°). Since the PM10 data can contain a considerable number of outliers, a 2-D Gaussian smoothing kernel with a standard deviation of 0.5 and logarithmization (to reduce skewness) was applied before clustering.

### Clustering

To cluster the PM10 time series, the k-means clustering method has been used, which identifies similar instances and assigns them to clusters^23^. Since the interest was to identify similar PM10 time series, the correlation distance was used as a measure of similarity. The k-means clustering algorithm maximizes the distance between different clusters and minimizes the distances within a cluster. In other words, the algorithm finds as many different clusters as possible and maintains the similarity within the clusters. The optimal number of clusters was determined using the silhouette score [-1, 1], which measures how similar an object is to its own cluster compared to other clusters. In this method, the average silhouette of the observations for a different number of clusters is calculated and the optimal number of clusters is determined by the maximum of the average silhouette, as a higher silhouette score indicates that the clusters have a high internal similarity and are also well separated from each other.

### Calculating trends and temporal shifts

The average time series of each cluster was used to calculate the long-term and short-term trend and to analyze the seasonal cycles. For this purpose, the method of singular spectrum analysis (SSA) was applied, which detects trends in a data vector^24–27^. SSA is a non-parametric spectral estimation method for time series that can effectively decompose and reconstruct signals. SSA assumes an additive decomposition of the data (A) such that A = LT + ST + R, where LT stands for the long-term trend, ST for the short-term trend and R for reminder. The basic SSA steps are decomposition and reconstruction, and both steps contain two further sub-steps. The decomposition step includes time-delayed embedding (to obtain the trajectory matrix) and singular value decomposition. Diagonal averaging and grouping, which are both part of the reconstruction step, are used to obtain reconstructed time series and categorize them into three classes: long-term trend, short-term trend (oscillations, seasonal cycles), and reminder or noise.

In order to calculate the temporal shift of the seasonal cycles (short-term trend) on the regional out-grid domain (-25 W/45E/30 N/70 N), the extreme values (local maxima and minima) were first determined in each grid. The first extreme value found in the period 2013–2022 was then used as a reference date. This allowed us to calculate the temporal shifts of the extreme values in relation to the first analyzed year 2013. In a second step, the difference in days between the respective extreme value and the first extreme value was calculated. Non-significant trends (*p* > 0.05) and when the difference between maximum and minimum was less than 3 µg m^− 3^ (to exclude rather small fluctuations) were set to zero.

### Software

All analyzes, with the exception of the remapping, which was carried out using the Climate Data Operators (CDO) software^28^, were performed in MATLAB^29^. The following built-in MATLAB functions *imgaussfilt*,* kmeans*,* trenddecomp* and *findpeaks* were used for 2D data smoothing, clustering, trend decomposition and searching for extreme values, respectively.

## Results and discussion

### Overlap of PM10 spatial patterns and clime zones

**Fig. 1:**
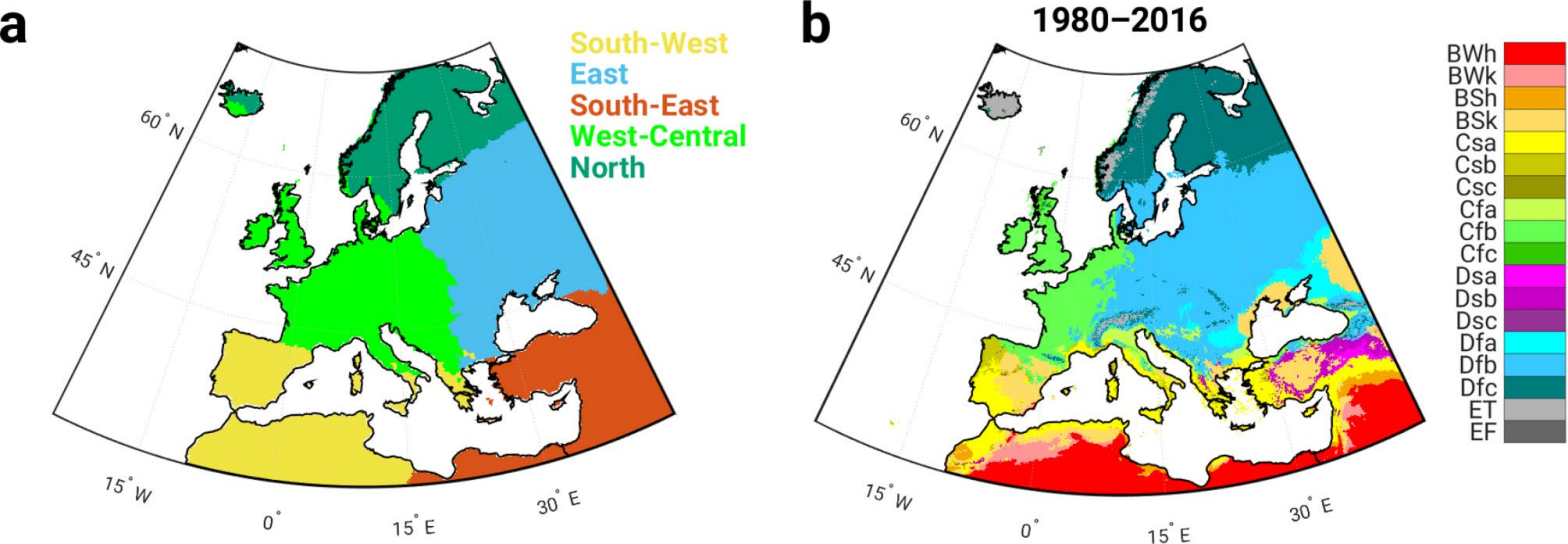
Cluster analysis and Köppen-Geiger climate zones. **(a)** five PM10 clusters, **(b)** present Köppen-Geiger climate zones^7^. The extended description of the color scheme of the Köppen-Geiger climate classes can be found on Supplementary Fig. 2.

In this study, the silhouette method was used to determine the optimal number of clusters. This is the point at which the average silhouette value reaches the local maximum value (Supplementary Fig. 1). Thus, by applying the unsupervised learning technique k-means clustering, we obtain the following five clusters in the analyzed out-grid domain (Fig. 1a): (i) the southwestern cluster, which includes the Iberian Peninsula and the northern part of Africa (Morocco and Algeria), (ii) the eastern cluster (Romania, Ukraine, Baltic countries, Belarus and eastern Russia), (iii) the southeastern cluster (Turkey and Syria), (iv) the west-central cluster (UK, France and Germany) and (v) the northern cluster (Norway, Sweden and Finland).

**Fig. 2:**
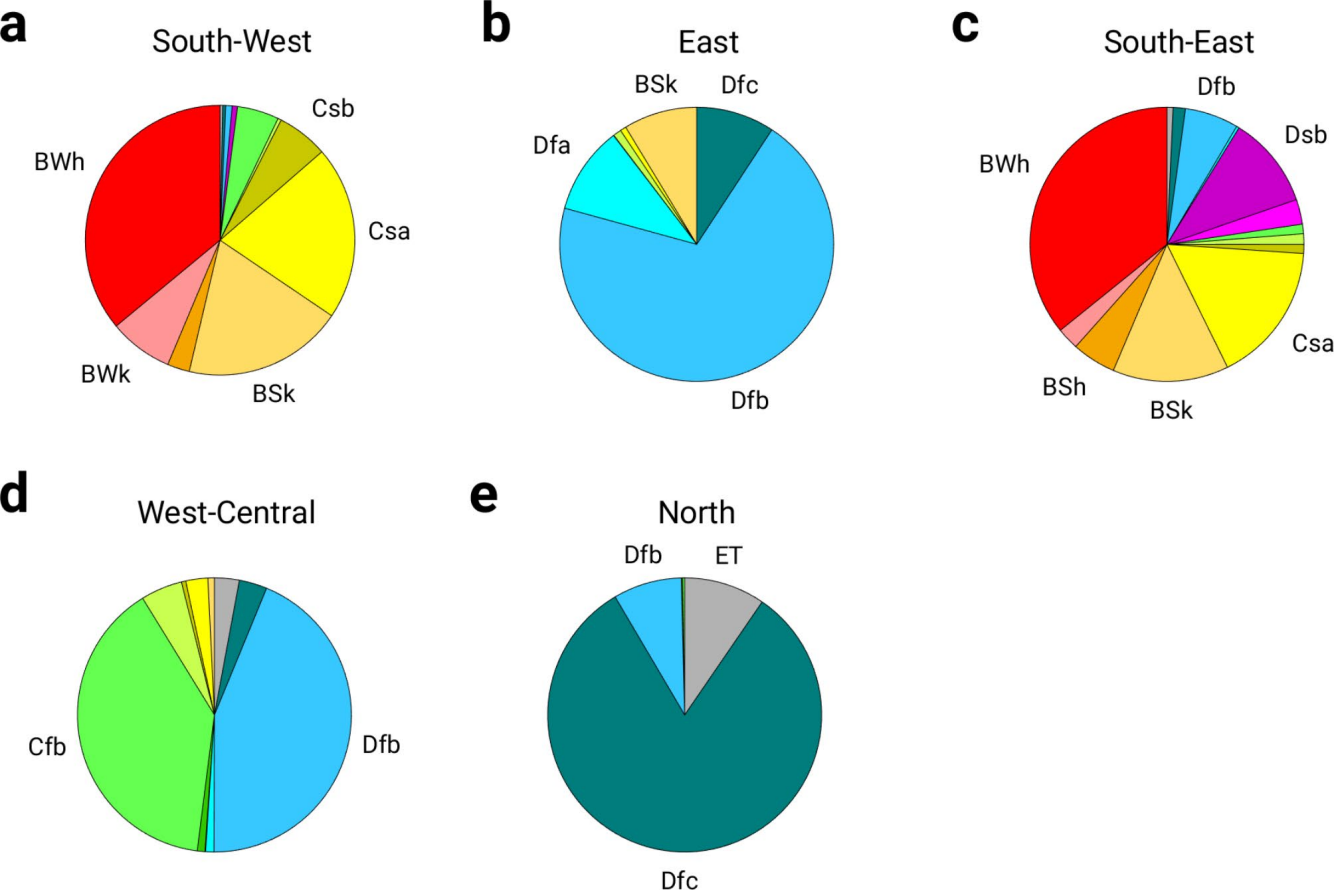
Pie diagram of the climate zones for individual PM10 clusters. The distribution of the present Köppen-Geiger climate zones for **(a)** the southwestern, **(b)** the eastern, **(c)** the southeastern, **(d)** the west-central and **(e)** the northern PM10 cluster. The extended description of the color scheme of the Köppen-Geiger climate classes can be found on Supplementary Fig. 2.

An analysis of the mapped PM10 clusters and the present Köppen-Geiger climate zones shows a good spatial overlap (Fig. 1). The southwestern cluster overlaps with hot arid (BWh), cold arid (BSk) and temperate climate with dry-hot summers (Csa) (Fig. 2a); the eastern cluster corresponds to cold arid (BSk) and cold continental climate (Dfa, Dfb, Dfc) (Fig. 2b); the southeastern cluster includes hot and cold arid (BWh, BSk), temperate climate with dry-hot summers (Csa) and cold continental climate (Dfb, Dsb) (Fig. 2c); the west-central cluster corresponds to the temperate oceanic climate (Cfb) and the cold continental climate (Dfb) (Fig. 2d); the northern cluster overlaps well with the cold continental (Dfb, Dfc) and polar (ET) climate regions (Fig. 2e).

**Fig. 3:**
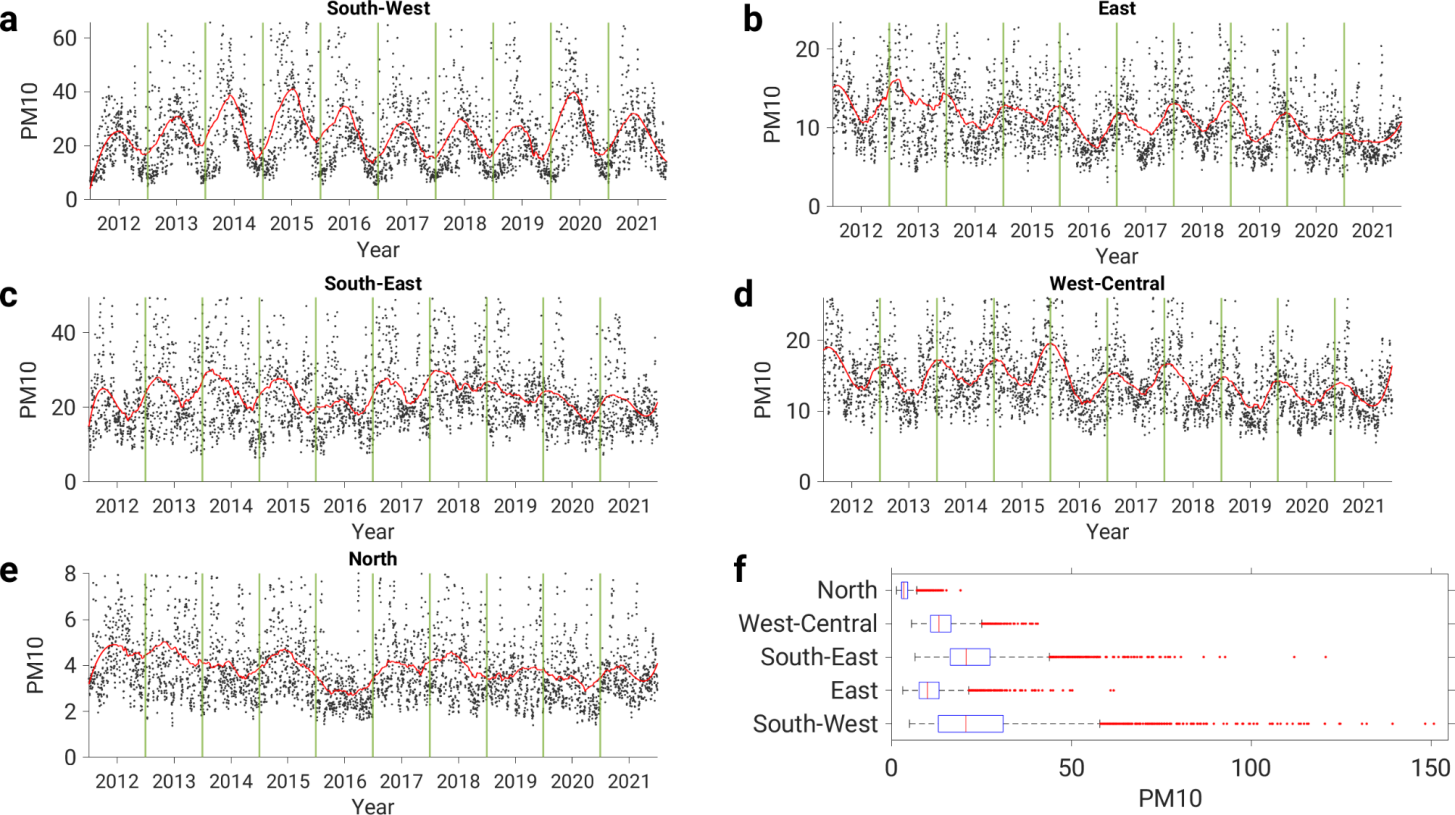
Averaged PM10 time series of five clusters, **(a)** south-west, **(b)** east, **(c)** south-east, **(d)** west-central and **(e)** northern cluster. The black dots represent the daily PM10 concentrations, while the read line represents the smoothed data. **(f)** Box-plot of PM10 concentration (µg m^− 3^) for each cluster.

Figure 3 shows the averaged PM10 time series for each cluster, and we can see clear differences between them, both in terms of the level of PM10 concentrations and seasonality (Fig. 3). The highest PM10 concentrations were clearly found in the southwestern and southeastern clusters and the lowest concentrations in the northern cluster (Fig. 3f). The mean PM10 concentration for the southwestern, eastern, southeastern, western central and northern clusters was 25 µg m^− 3^, 11 µg m^− 3^, 23 µg m^− 3^, 15 µg m^− 3^ and 4 µg m^− 3^, respectively. Furthermore, the lowest correlation (-0.21) was found between the PM10 time series of the southwestern and eastern clusters (Supplementary Fig. 3), while the highest correlation (0.34) was observed between the eastern and western-central clusters. In general, the pairwise correlation between the PM10 time series shows a rather low correlation (Supplementary Fig. 3), which indicates that the clusters show quite different seasonal fluctuations.

**Fig. 4:**
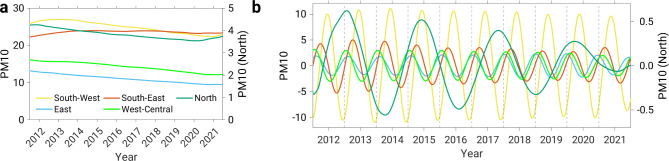
PM10 trends of five clusters. **(a)** Long-term trend and **(b)** short-term trend. The left y-axis of both diagrams shows the PM10 values of the southeastern, eastern, southwestern and west-central clusters, while the right y-axis shows the PM10 values of the northern cluster.

In order to additionally examine the long-term and short-term trends of the PM10 time series, the Singular Spectrum Analysis was applied to the PM10 time series. Figure 4a shows the long-term trend of the PM10 cluster time series. All clusters, with the exception of the southeastern cluster, show decreasing PM10 concentrations in the period 2012–2023. The southeastern cluster shows a slight increase in 2012 and 2013, after which no positive or negative trend can be seen from 2014 to 2022. These results are quite promising and confirm that PM10 concentrations in Europe have generally shown a negative trend over the last 10 years^30–32^. The reason for the negative trend in PM10 concentrations in Europe is mainly due to the reduction in primary emissions (15%) and precursor emissions (from 5 to 50%). The decrease in emissions was achieved by controlling and applying measures to reduce emissions in the industrial, energy and transport sectors^30,33^.

The short-term trend shows a pronounced seasonal variability of PM10 concentrations for most of Europe (Fig. 4b). In both the southeastern and southwestern clusters, the short-term trend shows high PM10 concentrations in spring and summer, i.e. during the warm season. In contrast, in the west-central and eastern clusters, the highest PM10 values are seen in the cold season. The short-term trends in PM10 levels thus show that the cold season is not necessarily associated with high PM10 concentrations. Note that there is no annual period in the northern cluster. Another observed difference between the short-term PM10 trends is the amplitude. The northern cluster has the smallest amplitude (∼ 1 µg m^− 3^). The largest amplitude or annual oscillation of PM10 values is observed for the southwestern cluster, where the difference between maximum and minimum values is ∼ 20 µg m^− 3^, while the oscillations in the southeastern, west-central and eastern clusters are twice as large at ∼ 10 µg m^− 3^.

The clustering of the PM10 time series revealed that there is a spatial overlap between the defined PM10 clusters and the Köppen-Geiger climate zones. PM10 concentrations are higher in arid climate zones than in temperate and cold climate zones. A greater amplitude, i.e. the difference between the seasonal maximum and minimum values, was also observed in arid climate zones than in cold and polar climate zones. Furthermore, seasonality is directly related to the climatic zones, for which it is characteristic that in temperate and cold climates high PM10 levels occur in the cold season, while in arid climates the situation is reversed.

### Temporal shifts of seasonal extremes

**Fig. 5:**
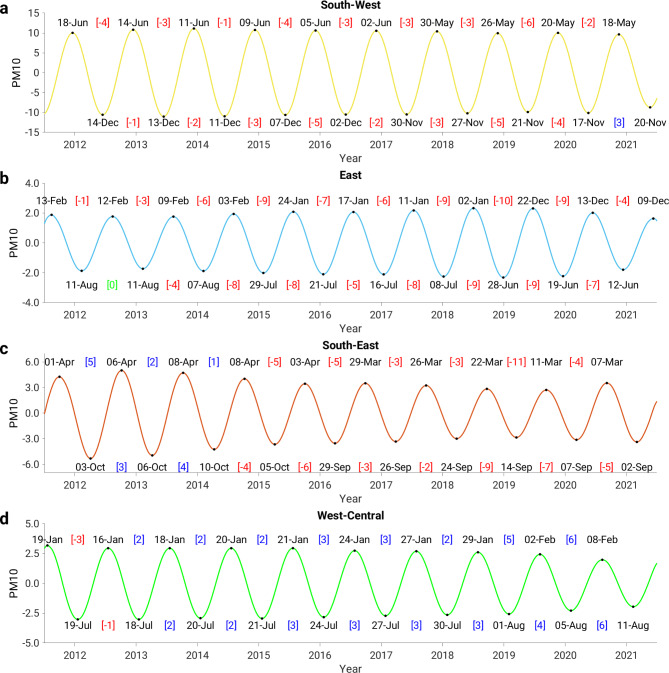
The temporal shift of PM10 maxima and minima values. Seasonal cycle (short-term trend) with labeled dates of (local) maximum and minimum PM10 values for **(a)** the southwestern, **(b)** the eastern, **(c)** the southeastern and **(d)** the west-central PM10 cluster. The temporal shift in days is given in square brackets, where red stands for negative (earlier), blue for positive (later) and green for no shift.

The analysis of short-term trends presented in the previous section has shown that four out of five clusters have an annual cycle. The short-term trend takes the form of a sinusoidal wave with two extremes per year, a maximum and a minimum (Fig. 5a-d). Note that an extreme value is either a local minimum or a local maximum. The short-term trend shows that the maximum and minimum PM10 concentrations in the southwestern and eastern clusters have shifted to earlier points in time in the period 2013–2022 (Fig. 5a, b). The temporal shift in the southwestern and eastern clusters was about − 3 and − 6 days/year, respectively. In the southwestern cluster, the PM10 maximum occurs earlier (shift from mid-June to mid-May, Fig. 5a), while in the eastern cluster there is a shift from mid-February to mid-December (Fig. 5b). Similarly, the minimum values in the southwestern and eastern clusters were also shifted to earlier dates (Fig. 5a, b). The southeastern cluster also shows a shift of maximum and minimum PM10 concentrations to earlier dates (Fig. 5c); however, the shift is not as evident as in the southwestern and eastern clusters. We can see a shift to later dates in the first three years and to earlier dates in the last five years of the period 2013–2022.

In contrast, the maximum and minimum values in the west-central cluster have shifted to later dates (Fig. 5d). The PM10 maximum value is thus reached later and shifts from January to February, while the minimum value shifts from July to August.

**Fig. 6:**
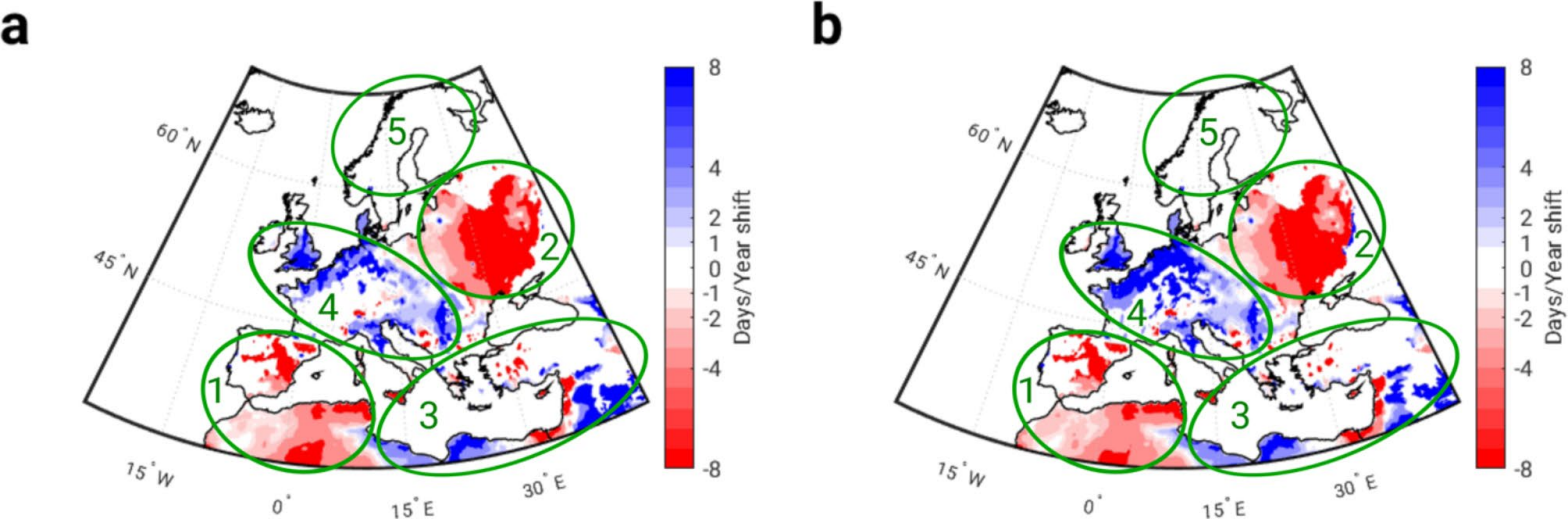
The temporal displacements of PM10 maximum and minimum values. **(a)** The shifts (days/year) of the maximum and **(b)** the minimum values. Regions 1, 2, 3, 4 and 5 marked with green ellipses correspond to the southwestern, eastern, southeastern, west-central and northern clusters, respectively. Red indicates a negative shift (earlier), blue a positive shift (later) and white a non-significant trend (*p* > 0.05).

In addition, the temporal shift of PM10 maxima and minima was investigated in each grid of the regional out-grid domain. Figure 6 clearly shows the regions that differ in the direction of the temporal shift (later/earlier) and in the magnitude - days per year. The results shown on the map confirm a strong temporal shift (later) of PM10 maxima and minima in Eastern (region 2) and Southwestern Europe (region 1), while the UK, part of Central Europe and the Balkans (region 4) show a shift to earlier dates.

Regions 3 and 5 correspond to the southeastern and northern clusters respectively. These two regions show no clear temporal shift. In northern Europe, the trend is not significant, or the seasonal variations were rather small (< 3 µg m^− 3^), while in the south-eastern part of Europe there are scattered patches of negative (earlier) and positive shifts (later).

This analysis has shown that the timing of extreme PM10 concentrations is shifting to earlier or later dates in most parts of the European continent over the period studied (2012 to 2023). The temporal shift of the maximum and minimum values varies both in terms of the temporal direction and the extent. The most significant shift occurred in the eastern part of Europe, where the extreme values shifted by two months (∼ 60 days). The analysis of the temporal shifts also confirmed the existence of two clusters in the central part of Europe (at latitude 50^O^N), as the short-term trend shows a temporal shift in the opposite direction. Indeed, the comparison between the seasonal cycles in the west-central and eastern clusters shows that the extremes have reversed in the period 2013–2022. For example, the maximum (minimum) in the west-central cluster shifted from mid-January (mid-July) to mid-February (mid-August), see Fig. 5d, while in the eastern cluster it shifted from mid-February (mid-August) to mid-December (mid-June), see Fig. 5b.

It should be noted that the temporal shifts in extreme PM10 concentrations are likely not the result of climate change on a longer, multi-decade time scale, such as the timing of floods^34^, the storm surge season^35^, the annual cycle of surface temperature^36^, or summer lake temperatures^37^. In the studies mentioned, data analysis shows that the shifts are of the order of a few days per decade, while the seasonal shifts in PM10 extremes show up to two-month shifts per decade. However, a longer data period (∼ 50 years) would be required to investigate a possible relationship between the timing of extreme PM10 concentrations on a time scale of several decades.

### PM10 concentration and future clime zones

**Fig. 7:**
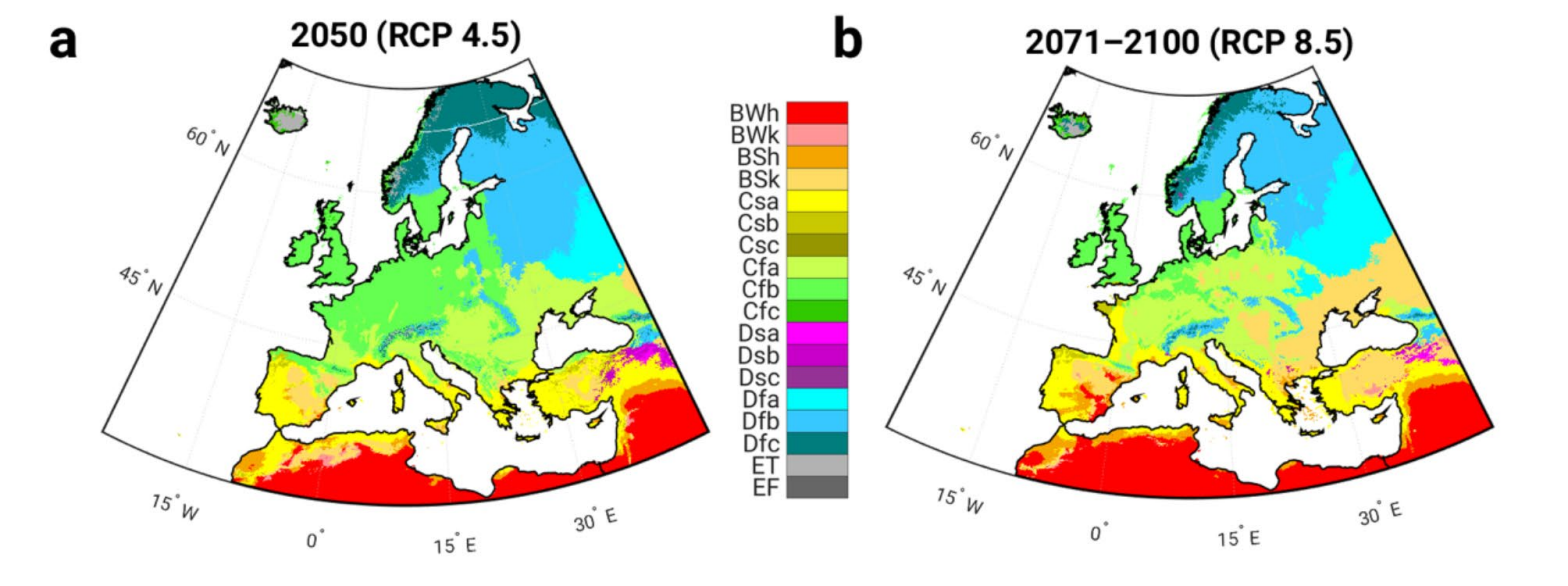
Köppen-Geiger climate zones. **(a)** Future map (2050) - scenario RCP4.5, **(b)** future map (2071–2100) - scenario RCP8.5. The extended description of the color scheme of the Köppen-Geiger climate classes can be found on Supplementary Fig. 1.

The relationship between possible shifts (expansion, contraction) of climate zones in the future and the knowledge about the overlap of PM10 clusters and climate zones gives us the possibility to qualitatively estimate possible changes in PM10 concentrations. The comparison between the present state (Fig. 1b) and the future climate zones display a poleward movement of certain climate zones^2,3,5–7,9^. The future map (2071–2100 - scenario RCP8.5) demonstrate that the boreal climate zone will almost disappear (Fig. 7b), the cool continental climate region will shift northwards and be replaced by the temperate climate zone. With the poleward movement of the arid climate zones, we can therefore expect an increase in PM10 concentrations at higher latitudes. Obviously, a smaller shift in climate zones was projected for 2050 under RCP 4.5 (Fig. 7a) than for 2071–2100 under RCP 8.5 (Fig. 7b). Although the change is smaller, the pattern of change is similar, meaning that climate zones C (temperate), D (continental) and E (polar) in particular shift northwards (Fig. 7a). The polar climate zone is replaced by the continental climate zone and the continental climate zone by the temperate climate zone, which could also lead to higher PM10 concentrations.

More detailed displacements (expansion/shrinkage) of the climate zones from the present to the future (2071–2100 - scenario RCP8.5) in the individual clusters and the corresponding PM10 concentrations can be found in the Supplementary Figs. 4–6. The two southern clusters show less extensive changes in the climate zones compared to the west-central and eastern clusters. The greater expansion and contraction of the climate zones can obviously be observed in the northern cluster.

The climate changes in the southwestern cluster show a further expansion of the arid (BWh, BSh) and a shrinkage of the BWk, BSk and Cfb climate zones (Supplementary Fig. 4a). A similar trend can also be seen for the southeastern cluster, which shows an additional expansion of the BWh and a shrinkage of the Dsb climate zones (Supplementary Fig. 4b). For both clusters, we can therefore conclude that the areas with a lower PM10 concentration will tend to shrink, while the areas, especially the BWh zone, which has the highest PM10 concentration, will tend to expand in the future (Supplementary Fig. 4c, d).

In the case of the eastern cluster, we can observe a decrease in the Dfb and Dfc climate and an increase in the BSk climate (cold, semi-arid), the Cfa climate (hot and humid summers, mild cool winters) and the Dfa climate (cold, no dry season, hot summer) (Supplementary Fig. 5a). Similar applies to the west-central cluster: Dfb decreases and Cfa increases (Supplementary Fig. 5b). The PM10 values of the expanding climate zones BSk, Cfa and Dfa show higher PM10 values than the climate zones Dfb and Dfc, which tend to shrink (Supplementary Fig. 5c, d).

As already mentioned, the northern cluster is exposed to the strongest changes in climate zones. Obviously, we can recognize a poleward movement of the climate zones, with the climate zone Dfc tending to shrink while the climate zone Dfb grows (Supplementary Fig. 6a). In addition, climate zone ET (polar, tundra) tends to shrink, while climate zone Cfb (temperate, no dry season, warm summer) expands. When comparing the PM10 concentrations corresponding to the climate zones, we see that the climate zones with higher PM10 concentrations tend to become larger and the climate zones with lower PM10 concentrations tend to occupy a smaller area (Supplementary Fig. 6b).

PM10 concentrations are the result of natural and anthropogenic emissions as well as various meteorological effects that are characteristic of the respective climate zone. The scavenging of PM10 is related to climate, as longer periods without rain may reduce the wet deposition. In addition, changes in the length of seasons, such as summer and winter, can also significantly influence annual PM10 cycles. Over the last ten years, a downward trend in PM10 concentrations has been observed in Europe^30–32^, which was also confirmed in this study. However, due to climate change and the spread of climate zones that currently have higher PM10 concentrations, the negative PM10 trend could reverse to a positive one, especially in northern Europe.

## Conclusion

The Köppen-Geiger classification and its usefulness not only for soil types and biomes, but also for the identification of air pollution, more precisely of common spatial patterns of PM10 in Europe was presented. Unsupervised clustering of PM10 time series revealed clusters that show a good spatial overlap with the main Köppen-Geiger climate classes. This is not unexpected as climate is related to precipitation, temperature, wind, weather patterns and natural emissions, all of which have a strong influence on PM10 concentrations. Five PM10 clusters have been identified on the European continent: southwestern, southeastern, west-central, eastern and northern. The long-term trend showed a decrease in PM10 levels for all clusters in the period 2013–2022, while the analysis of the seasonal cycles shows a different seasonal variability and a shift in the timing of the maxima and minima of PM10 concentrations in the period 2013–2022.

However, knowing the spatial overlap between climate zones and clustered PM10 time series allows us to qualitatively estimate possible changes in air pollution. If the spatial overlap between PM10 and climate zones remains in the future and climate changes occur, we can expect higher PM10 concentrations due to the poleward shift of climate zones, especially in the northern part of Europe. In addition, the regime of high and low concentrations could also change, as it differs when we compare temperate and cold with arid climates.

The limitation of this study is that possible future anthropogenic emissions were not considered. It should be emphasized that several other studies that have predicted future particulate matter concentrations under different climate change scenarios have also used current (year ∼ 2000) anthropogenic emissions. A better understanding of the relationship between climate zones and air pollution and the availability of high-quality climate maps for the future can therefore be used as a preliminary estimate of the change in PM10 concentrations and to assess the potential impact on public health and mortality.

## Electronic supplementary material

Below is the link to the electronic supplementary material.

Supplementary Material 1

## Data Availability

The datasets generated and analyzed during the current study are available on request from the corresponding author.
