# Supplementary material for: The relationship between climate classes and particulate matters over Europe
Source: Sci Rep. 2024 Nov 21;14:28821. doi: 10.1038/s41598-024-80365-7 (PMC11582561; doi:10.1038/s41598-024-80365-7)
Supplement: Supplementary file 1 — Supplementary Material 1 [file 41598_2024_80365_MOESM1_ESM.pdf]

Supplementary Material

The relationship between climate classes and particulate matters over Europe

Jure Pražnikar[1*]
[1]Faculty of Mathematics, Natural Sciences and Information Technologies, University of Primorska, Slovenia.

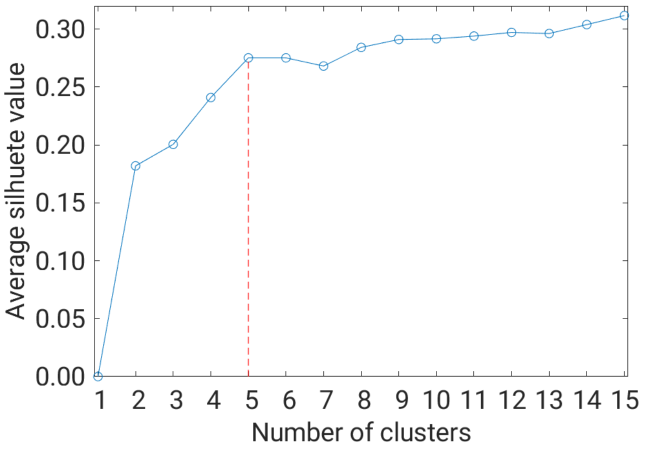

Supplementary Figure 1: Optimal number of clusters. Average silhouette value versus number of clusters.

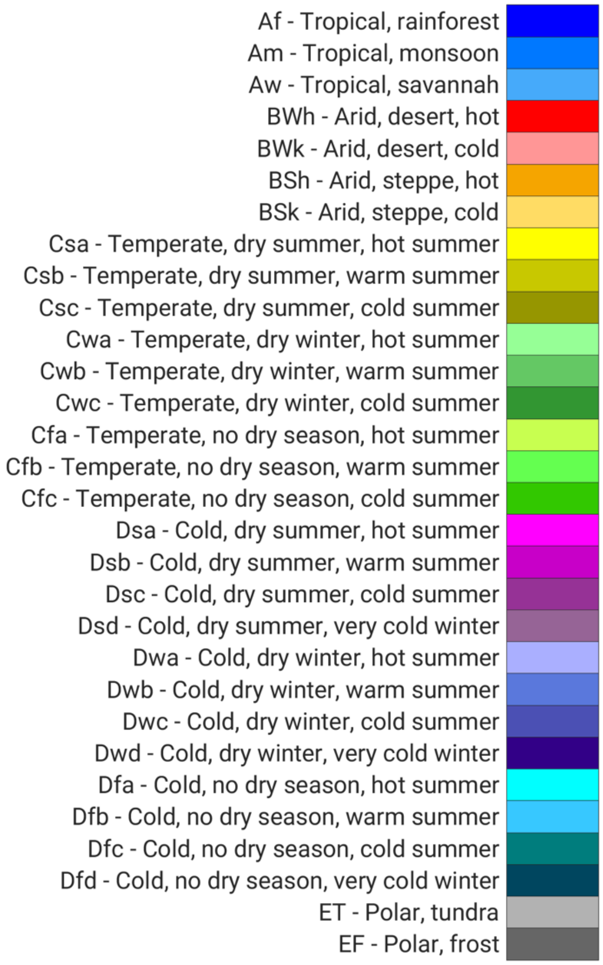

Supplementary Figure 2: Color scheme of Köppen-Geiger climate classes.

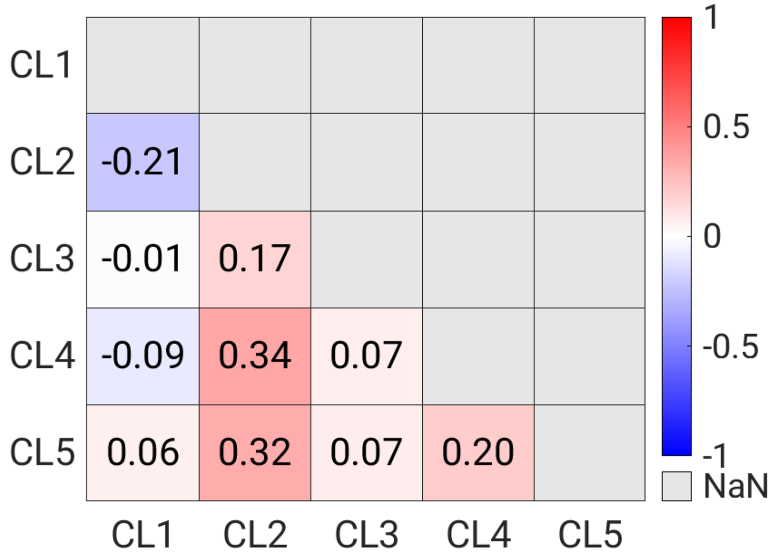

Supplementary Figure 3: The pairwise correlation between the PM10 time series. The labels CL1, CL2, CL3, CL4 and CL5 correspond to the southwestern, eastern, southeastern, west-central and northern clusters, respectively.

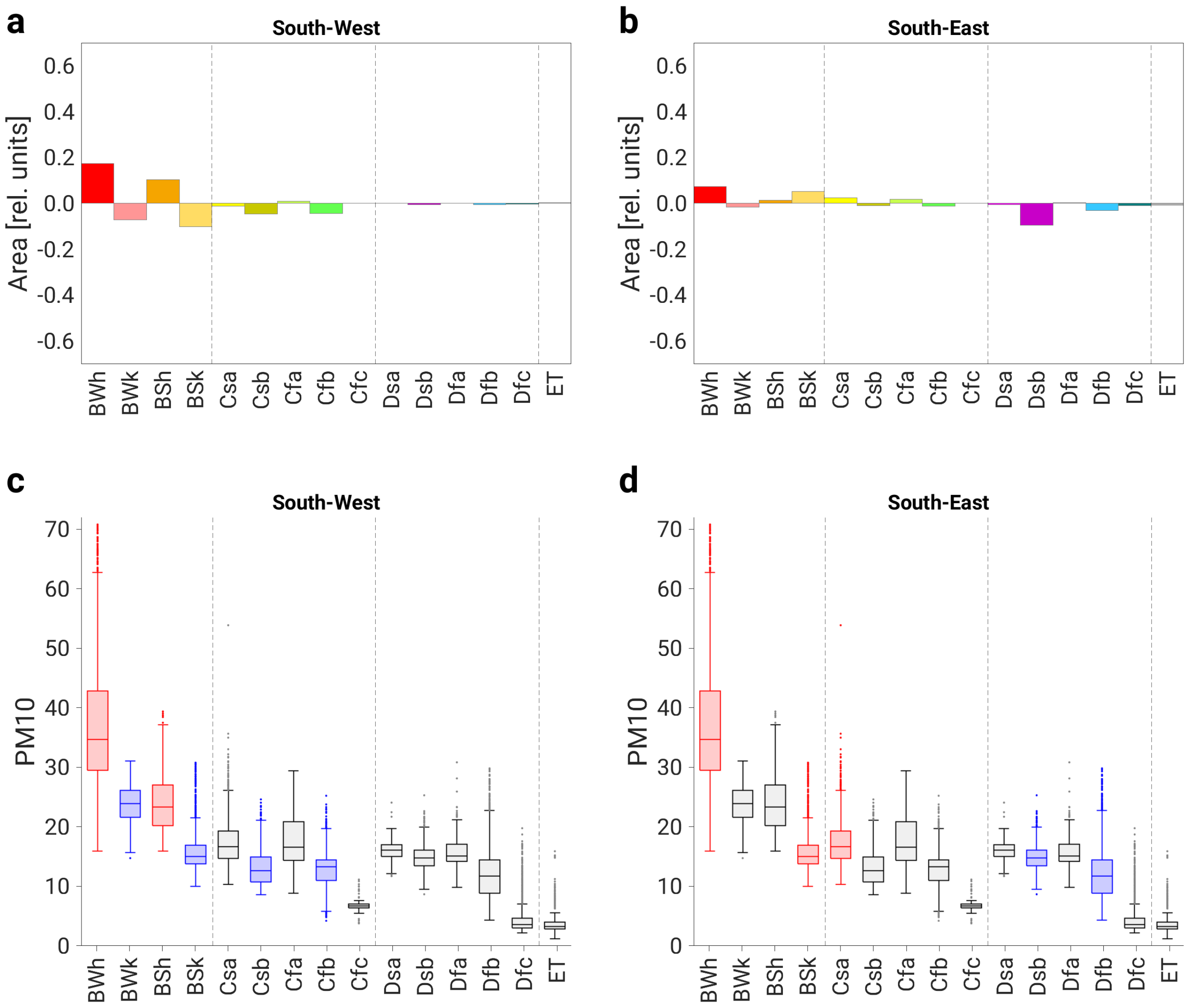

Supplementary Figure 4: PM10 concentrations and the change in climate zones in the southwestern and southeastern clusters. (a, b) The bar charts show the relative change between the future (2071-2100 under RCP 8.5) and present climate zones. (c, d) Boxplots of PM10 concentrations for the present climate zones in the regional out-grid domain (-25W/45E/30N/70N). The red color indicates the expansion of the respective climate zone, while the blue color indicates the contraction. Note that boxplots where the absolute value of the difference between present and future climate zones is less than 2% are displayed in light gray. The extended description of the color scheme of the Köppen-Geiger climate classes can be found on Supplementary Fig. 2.

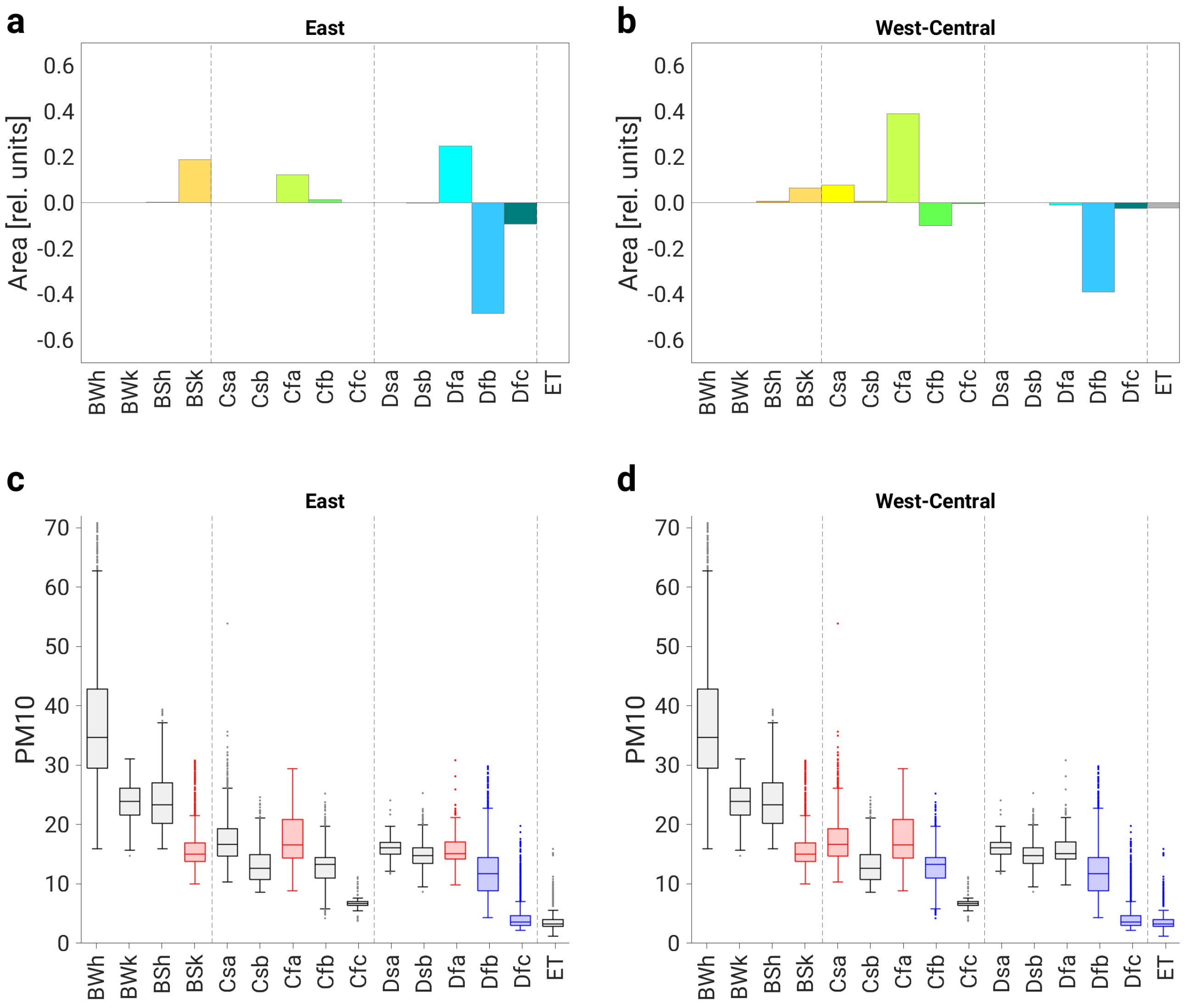

Supplementary Figure 5: PM10 concentrations and the change in climate zones in the eastern and west-central clusters. (a, b) The bar charts show the relative change between the future (2071-2100 under RCP 8.5) and present climate zones. (c, d) Boxplots of PM10 concentrations for the present climate zones in the regional out-grid domain (-25W/45E/30N/70N). The red color indicates the expansion of the respective climate zone, while the blue color indicates the contraction. Note that boxplots where the absolute value of the difference between present and future climate zones is less than 2% are displayed in light gray. The extended description of the color scheme of the Köppen-Geiger climate classes can be found on Supplementary Fig. 2.

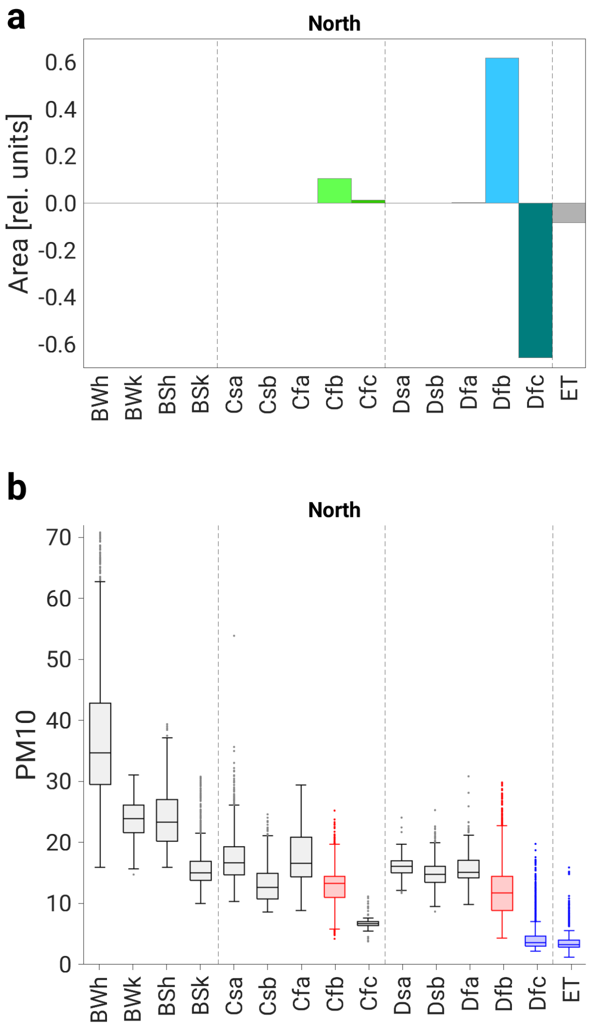

Supplementary Figure 6: PM10 concentrations and the change in climate zones in the northern cluster. (a) The bar charts show the relative change between the future (2071-2100 under RCP 8.5) and present climate zones. (b) Boxplots of PM10 concentrations for the present climate zones in the regional out-grid domain (-25W/45E/30N/70N). The red color indicates the expansion of the respective climate zone, while the blue color indicates the contraction. Note that boxplots where the absolute value of the difference between present and future climate zones is less than 2% are displayed in light gray. The extended description of the color scheme of the Köppen-Geiger climate classes can be found on Supplementary Fig. 2.
